# Functional mapping of reaction norms to multiple environmental signals through nonparametric covariance estimation

**DOI:** 10.1186/1471-2229-11-23

**Published:** 2011-01-26

**Authors:** John S Yap, Yao Li, Kiranmoy Das, Jiahan Li, Rongling Wu

**Affiliations:** 1 Department of Statistics, University of Florida, Gainesville, FL 32611 USA; 2 Department of Statistics, West Virginia University, Morgantown, WV 26506, USA; 3 Center for Statistical Genetics, Pennsylvania State University, Hershey, PA 17033, USA; 4 Center for Computational Biology, Beijing Forestry University, Beijing 100083, PR China

## Abstract

**Background:**

The identification of genes or quantitative trait loci that are expressed in response to different environmental factors such as temperature and light, through functional mapping, critically relies on precise modeling of the covariance structure. Previous work used separable parametric covariance structures, such as a Kronecker product of autoregressive one [AR(1)] matrices, that do not account for interaction effects of different environmental factors.

**Results:**

We implement a more robust nonparametric covariance estimator to model these interactions within the framework of functional mapping of reaction norms to two signals. Our results from Monte Carlo simulations show that this estimator can be useful in modeling interactions that exist between two environmental signals. The interactions are simulated using nonseparable covariance models with spatio-temporal structural forms that mimic interaction effects.

**Conclusions:**

The nonparametric covariance estimator has an advantage over separable parametric covariance estimators in the detection of QTL location, thus extending the breadth of use of functional mapping in practical settings.

## Background

The phenotype of a quantitative trait exhibits *plasticity *if the trait differs in phenotypes with changing environment [1-7]. Such environment-dependent changes, also called *reaction norms*, are ubiquitous in biology. For example, thermal reaction norms show how performance, such as caterpillar growth rate [8] or growth rate and body size in ectotherms [9], varies continuously with temperature [10]. Another example is the flowering time of *Arabidopsis thaliana *with respect to changing light intensity [11]. However, QTL mapping of reaction norms is difficult to model because of the inherent complexity in the interplay of a multitude of factors involved. An added difficulty is in their being "infinite-dimensional" as they require an infinite number of measurements to be completely described [12]. Wu et al. [13] proposed a functional mapping-based model which addresses the latter difficulty by using a biologically relevant mathematical function to model reaction norms. The authors considered a parametric model of photosynthetic rate as a function of light irradiance and temperature and studied the genetic mechanism of such process. They showed through simulations that in a backcross population with one or two-QTLs, their method accurately and precisely estimated the QTL location(s) and the parameters of the mean model for photosynthesis rate. For a backcross population with one QTL, the mean model consists of two surfaces that describe the photosynthetic rate of two genotypes. However, in their model, they assumed the covariance matrix to be a Kronecker product of two AR(1) structures, each modeling a reaction norm due to one environmental factor. This type of covariance model is said to be *separable*. Although computationally efficient because of the minimal number of parameters to be estimated, this model only captures separate reaction norm effects but fails to incorporate interactions. A more general approach is therefore needed.

In the context of longitudinal data, Yap et al. [14] proposed a nonparametric covariance estimator in functional mapping. It was nonparametric in the sense that the covariance matrix has an unconstrained set of parameters to be estimated and not the usual distribution-free sense in nonparametric statistics. This estimator can be obtained by employing a modified Cholesky decomposition of the covariance matrix which yields component matrices whose elements can be interpreted and modeled as terms in a regression [15]. A penalized likelihood procedure is used to solve the regression with either an *L*_1 _or *L*_2 _penalty [16]. Penalized likelihood in regression is a technique used to obtain minimum mean squared error (MSE) of estimated regression coefficients by balancing bias and variance. *L*_1 _or *L*_2 _penalties, which are functions of the regression covariates, are included in a regression model in order to shrink coefficients towards estimates with minimum MSE. In the case of the *L*_1 _penalty, some of the coefficients are actually shrunk to zero. Thus, with the *L*_1 _penalty, a more parsimonious regression model is obtained. The use of penalized likelihood with *L*_1 _or *L*_2 _penalties is particularly useful when there is multi-collinearity among the covariates in the regression i.e. when there are near linear dependencies or high correlations among the regressors or predictor variables. An iterative procedure is implemented by using the ECM algorithm [17] to obtain the final estimator. Through Monte Carlo simulations, this nonparametric estimator is found to provide more accurate and precise mean parameters and QTL location estimates than the parametric AR(1) form for the covariance model, especially when the underlying covariance structure of the data is significantly different from the assumed model.

The question of how to incorporate interaction effects in a model with multiple factors has not, to our knowledge, been thoroughly explored in the biology literature, especially in the context of genetic mapping that incorporates interactions of function-valued traits. The spatio-temporal literature, however, has a wealth of publications that developed more general models such as *nonseparable *covariance structures which are used to model the underlying interactions of random processes in the space and time domains (see [18,19]). A nonseparable covariance cannot be expressed as a Kronecker product of two matrices like separable structures can. The random processes being modeled may be the concentration of pollutants in the atmosphere, groundwater contaminants, wind speed, or even disposable household incomes. The main significance of the covariance in this context is in providing a better characterization of the random process to obtain optimal *kriging *or prediction of unobserved portions of it. It therefore seems natural to consider the utilization of nonseparable structures in the simulation and modeling of reaction norms that react to two environmental factors. More concretely, we consider the photosynthetic rate as a random process, and the irradiance and temperature as the spatial (one dimension) and temporal domains, respectively.

The remaining part of this paper is organized as follows: We first describe the functional mapping model proposed by Wu et al. [13] for reaction norms. Then, we formulate separable and nonseparable models used in spatio-temporal analyses and present a simulation study using some nonseparable structures. Lastly, the new model and its implications for genetic mapping are discussed. From hereon, the terms covariance matrix, covariance structure or covariance function are used interchangeably.

## Functional Mapping of Reaction Norms

### Reaction Norms: An Example

Wolf [20] described a reaction norm as a surface landscape determined by genetic and environmental factors. The surface is characterized by a phenotypic trait as a function of different environmental factors such as temperature, light intensity, humidity, etc., and corresponds to a specific genetic effect such as additive, dominant or epistatic [21]. At least in three dimensions, the features of the surface such as "slope", "curvature", "peak valley", and "ridge", can be described graphically to help visualize and elucidate how the underlying factors affect the phenotype.

An example of reaction norms that illustrate a surface landscape is *photosynthesis *[13], the process by which light energy is converted to chemical energy by plants and other living organisms. It is an important yet complex process because it involves several factors such as the age of a leaf (where photosynthesis takes place in most plants), the concentration of carbon dioxide in the environment, temperature, light irradiance, available nutrients and water in the soil. A mathematical expression for the rate of single-leaf photosynthesis, *P*, without photorespiration [22] is

(1) P=αI +Pm2θ−b2−4θαIPm2θ

where *b *= (*αI *+ *P*_*m*_, *θ *∈ (0,1) is a dimensionless parameter, *α *is the photochemical efficiency, *I *is the irradiance, and *P*_*m *_is the asymptotic photosynthetic rate at a saturating irradiance. *P*_*m *_is a linear function of the temperature, *T*

(2) $$P_{m}=\{\begin{array}{cc} P_{m}(20)P(T) & T\geq T*\\ 0 & T<T^{*}, \end{array}$$

where $P(T)=\frac{T-T^{*}}{20-T^{*}}$, *P*_*m*_(20) is the value of *P*_*m *_at the reference temperature of 20°C and T* is the temperature at which photosynthesis stops. T* is chosen over a range of temperatures, such as 5°C-25°C, to provide a good fit to observed data.

**Figure 1:**
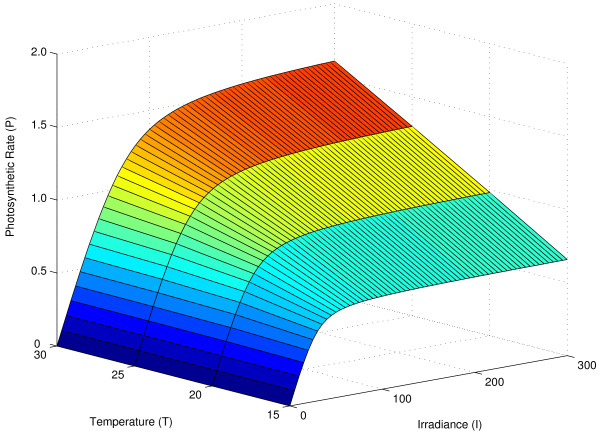
**Reaction norm surface of photosynthetic rate as a function of irradiance and temperature**. Model is based on equations (1) and (2) with parameters (*α*, *P*_*m*_(20), *θ*) = (0.02, 1, 0.9). Adapted from [13].

Wu et al. [13] studied the reaction norm of photosynthetic rate, defined by Eqs. (1) and (2), as a function of irradiance (*I*) and temperature (*T*). That is, the authors considered *P *= *P*(*I*, *T*). We assume that T* = 5 so that the reaction norm model parameters are (*α*, *P*_*m*_(20), *θ*). The surface landscape that describes the reaction norm of *P *(*I,T*), with parameters (*α, P_m_*(20), *θ*) = (0.02, 1, 0.9), is shown in Figure 1. As stated earlier, each reaction norm surface corresponds to a specific genetic effect. Thus, if a QTL is at work, the genetic effects produce different surfaces defined by distinct sets of model parameters corresponding to different genotypes.

### Likelihood

We consider a backcross design with one QTL. Extensions to more complicated designs and the two-QTL case, as in [13], are straightforward. Assume a backcross plant population of size *n *with a single QTL affecting the phenotypic trait of photosynthetic rate. The photosynthetic rate for each progeny *i *(*i *= 1, ..., *n*) is measured at different irradiance (*s *= 1, ..., *S*) and temperature (*t *= 1, ..., *T *) levels. This choice of variables is adopted for consistency in later discussions as we will be working with spatio-*t*emporal covariance models. The set of phenotype measurements or observations can be written in vector form as

(3) yi =[yi(1,1),...,yi(1,T),︸irradiance 1... ,[yi(S,1),...,yi(S,T)',︸irradiance S

The progeny are genotyped for molecular markers to construct a genetic linkage map for the segregating QTL in the population. This means that the genotypes of the markers are observed and will be used, along with the phenotype measurements, to predict the QTL. With a backcross design, the QTL has two possible genotypes (as do the markers) which shall be indexed by *k *= 1, 2. The likelihood function based on the phenotype and marker data can be formulated as

(4) $$L(\Omega )=\prod_{i=1}^{n}\left[\sum_{k=1}^{2}p_{k|i}f_{k}({\text{y}}_{i}|\Omega )\right]$$

where *p*_*k*|*i *_is the conditional probability of a QTL genotype given the genotype of a marker interval for progeny *i*. We assume a multivariate normal density for the phenotype vector y*_i _*with genotype-specific means

(5) μk =[μk(1,1),...,μk(1,T),︸irradiance 1... ,[μk(S,1),...,μk(S,T)',︸irradiance S

and covariance matrix Σ = cov(y*_i_*).

### Mean and Covariance Models

The mean vector for photosynthetic rate in (5) can be modeled using equations (1) and (2) as

(6) $$\begin{array}{c} \mu_{k}(s,\;t)\;=\frac{\alpha_{k}s\;+P_{mk}}{2\theta_{k}}\\ -\frac{\sqrt{b_{k}^{2}-4\theta_{k}\alpha_{k}sP_{mk}}}{2\theta_{k}} \end{array}$$

Where *b_k _= α_k_s *+ *P*_*mk*_,

(7) $$P_{mk}(t)=\{\begin{array}{cc} P_{mk}(20)P(t) & t\geq T*\\ 0 & t<T^{*} \end{array}$$

$P(t)=\frac{t-T^{*}}{20-T^{*}}$ and *k *= 1, 2.

Wu et al. [13] used a separable structure (Mitchell et al., 2005) for the *ST *× *ST *covariance matrix Σ as

(8) $$\Sigma_{AR(1)}=\Sigma_{1}⊗\Sigma_{2}$$

where Σ_1 _and Σ_2 _are the (*S×S*) and (*T×T*) covariance matrices among different irradiance and temperature levels, respectively, and ⊗ is the Kronecker product operator. Note that Σ_1 _and Σ_2 _are unique only up to multiples of a constant because for some |c| > 0, cΣ_1 _⊗ (1/c)Σ_2 _= Σ_1 _⊗ Σ_2_. Each of Σ_1 _and Σ_2 _is modeled using an AR(1) structure with a common error variance, σ^2^, and correlation parameters *ρ**_k _***(*k *= 1, 2):

(9) $$\Sigma_{k}=\sigma^{2}\left[\begin{array}{cccc} 1 & \rho_{k} & \cdots & \rho_{k}^{S-1}\\ \rho_{k} & 1 & \cdots & \rho_{k}^{S-2}\\ \vdots & \vdots & \ddots & \vdots \\ \rho_{k}^{S-1} & \rho_{k}^{S-2} & \cdots & 1 \end{array}\right]$$

Separable covariance structures, however, cannot model interaction effects of each reaction norm to temperature and irradiance. Thus, there is a need for a more general model for this purpose.

Yap et al. [14] proposed to use a data-driven nonparametric covariance estimator in functional mapping. The authors showed that using such estimator provides better estimates for QTL location and mean model parameters when compared to AR(1). Huang et al. [16] showed that the nonparametric estimator works well for large matrices. Functional mapping of reaction norms when there are two environmental signals necessitates the use of large covariance matrices that result from Kronecker products of smaller matrices. Here, we are interested in determining whether the nonparametric covariance estimator of Yap et al. [14] will still work well in this reaction norm setting.

It should be noted that unlike parametric models, e.g. AR(1), there are no parameters being estimated in the nonparametric covariance estimator. The entries of the matrix are determined based on the data. This is different from a model-dependent covariance matrix model with one parameter for each of its elements. Due to over-parametrization, such a model may not lead to convergence to yield reliable results.

Note that with (6)-(9), **Ω **= **Ω_1 _**∪ **Ω_2 _**in (4), where **Ω_1 _**= {*α*_1_, *P*_*m*1_(20), *θ*_1_, *σ*^2^, *ρ_1_*} and **Ω_1 _**= {*α*_2_, *P*_*m*2_(20), *θ*_2_, *σ*^2^, *ρ*_2_**}**. These model parameters may be estimated using the ECM algorithm [17], but closed form solutions at the CM-step are be very complicated. A more efficient method is to use the Nelder-Mead simplex algorithm [23] which can be easily implemented using softwares such as Matlab.

### Hypothesis Tests

The features of the surface landscape are important because they can be used as a basis in formulating hypothesis tests. Let *H*_0 _and *H*_1 _denote the null and alternative hypotheses, respectively. Then the existence of a QTL that determines the reaction norm curves can be formulated as

$$H_{0}:\alpha_{1}=\alpha_{2},P_{m1}(20)=P_{m}(20),\theta_{1}=\theta_{2},$$

versus

*H*_1 _: at least one of the equalities

above does not hold

This means that if the reaction norm curves are distinct (in terms of their respective estimated parameters), then a QTL possibly exists. The estimated location of the QTL is at the point at which the log-likelihood ratio obtained using the null and alternative hypotheses is maximal. Of course a slight difference in parameter estimates does not automatically mean a QTL exists. The significance of the results can be determined by permutation tests [24] which involves a repeated application of the functional mapping model on the data where the phenotype and marker associations are broken to simulate the null hypothesis of no QTL. A significance level is then obtained based on the maximal log-likelihood ratio at each application to infer the presence or absence of a QTL (see ref. [25] for more details). A procedure described in ref. [26] can be used to test the additive effects of a QTL. Other hypotheses can be formulated and tested such as the genetic control of the reaction norm to each environmental factor, interaction effects between environmental factors on the phenotype, and the marginal slope of the reaction norm with respect to each environmental factor or the gradient of the reaction norm itself. The reader is referred to Wu et al. [13] for more details.

## Spatio-Temporal Covariances

We investigate the use of parametric and nonseparable spatio-temporal covariance structures in functional mapping of photosynthetic rate as a reaction norm to the environmental factors irradiance and temperature. As stated earlier, the main idea is to model irradiance as a one-dimensional spatial variable and temperature as a temporal variable. The choice of which environmental signal is modeled as temporal or spatial is arbitrary. For more about spatio-temporal modeling, we refer the reader to [27,19].

### Basic Ideas, Notation, and Assumptions

We consider a real-valued spatio-temporal random process given by

(10) $$Y(s,\;t),\;(s,\;t)\in {\mathbb{R}}^{d}\times \mathbb{R},\;d\in {\text{ℤ}}^{+}$$

where observations are collected at coordinates

$$\left(s_{1},t_{1}),(s_{2},t_{2}),...,(s_{N},t_{N}\right)$$

to characterize unobserved portions of the process. This collection of coordinates are not necessarily ordered fixed levels of each trait. We will only be concerned with the case *d *= 1. Aside from those mentioned earlier, *Y *may also represent ozone levels, disease incidence, ocean current patterns or water temperatures. In our setting, *Y *represents photosynthetic rate.

If var (*Y*(*s*, *t*)) < ∞ for all (*s*, *t*) ∈ ℛ × ℛ, then the covariance, cov (*Y*(*s*, *t*), *Y*(*s *+ *u*, *t *+ *v*)), where *u *and *v *are spatial and temporal lags, respectively, exists. We assume that the covariance is *stationary *in space and time so that for some function *C*,

(11) cov (Y(s, t), Y(s+u, t+v))=C(u, v).

This means that the covariance function *C *depends only on the lags and not on the values of the coordinates themselves. Stationarity is often assumed to allow estimation of the covariance function from the data [18]. We also assume that the covariance function is *isotropic *which means that it depends only on the absolute lags and not in the direction or orientation of the coordinates to each other. The covariances considered in this paper are *positive (semi*-*) definite *as they satisfy the following condition: for any (*s*_1_, *t*_1_), ..., (*s_k _*, *t_k_*) ∈ ℛ × ℛ, any real coefficients *a*_1_, ..., *a_k_*, and any positive integer *k*,

(12) $$\sum_{i=1}^{k}\sum_{j=1}^{k}a_{i}a_{j}C(s_{i}-s_{j},\;t_{i}-t_{j})\geq 0$$

Note that *C*(*u*, 0) and *C*(0, *v*) correspond to purely spatial and purely temporal covariance functions, respectively.

In spatio-temporal analysis, the ultimate goal is optimal prediction (or kriging) of an un-observed part of the random process *Y*(*s*, *t*) using an appropriate covariance function model. We utilize a covariance model to calculate the mixture likelihood associated with functional mapping.

### Separable and Nonseparable Covariance Structures

#### Separable Covariance Structures

A covariance function *C*(*u, v*|*θ*) of a spatio-temporal process is *separable *if it can be expressed as

(13) $$C(u,\;v|\theta )=C_{1}(u|\theta_{1})C_{2}(v|\theta_{2})$$

where *C*_1_(*u***|***θ*_1_) and *C*_2_(*v***|***θ*_2_) are purely spatial and purely temporal covariance functions, respectively, and *θ *= (*θ*_1_, *θ*_2_)'. This representation implies that the observed joint process can be seen as a product of two independent spatial and temporal processes.

A more general definition for separability is as a Kronecker product (equation (8)). From equation (8), it can be shown that $\Sigma_{AR(1)}^{-1}=\Sigma_{1}^{-1}⊗\Sigma_{2}^{-1}$ and $|\Sigma_{AR(1)}|=|\Sigma_{1}{|}^{d_{2}}|\Sigma_{2}{|}^{d_{1}}$, where |·| denotes the determinant of a matrix; *d*_1 _and *d*_2 _are the dimensions of Σ_1 _and Σ_2_, respectively. This illustrates the computational advantage of using separable models in likelihood estimation where the inverse and determinant of the covariance matrix are calculated. For a large covariance matrix of dimension *UV*, its inverse can be calculated from the inverses of its Kronecker component matrices, Σ_1 _and Σ_2_, with dimensions *U *and *V*, respectively. Thus, the inversion of a 100 × 100 matrix, for example, may only require the inversion of two 10 × 10 matrices. A similar argument can be used for the determinant. Σ_*AR***(**1**) **_can be put in the form (13) as

(14) $$\begin{array}{l} C(u,\;v|\sigma^{2},\;\rho_{1},\;\rho_{2})\;=\;\sigma^{2}\rho_{1}^{u}\;\text{. }\sigma^{2}\rho_{2}^{v}\\ \;\;\;\;\;\;\;\;\;\;\;\;\;\;\;\;\;\;\;\;\;\;\;\;\;\;\;\;\;\;\;\;\;\;\;\;\;\;\;\;=\sigma^{4}\rho_{1}^{u}\rho_{2}^{v}, \end{array}$$

where *u *= 1, ..., *U *, *v *= 1, ..., *V*. Note that this model assumes equidistant or regularly spaced coordinates. Thus, two consecutive or closest neighbor coordinates will have the same correlation structure as another even if their respective distances are different. A more appropriate model might be

(15) $$C(u,\;v|\sigma^{2},\;\rho_{1},\;\rho_{2},\;a,\;b)=\sigma^{4}\rho_{1}^{u/a}\rho_{2}^{v/b}$$

where *a *and *b *are scale parameters. In this model, the scale parameters correct for the uneven distances between coordinates.

#### Nonseparable Covariance Structures

Here, we present some nonseparable covariance models that were derived in two different ways. The details of the derivation are omitted as they are rather complicated and lengthy.

The following nonseparable covariance models were derived by Cressie and Huang [18] using the Fourier transform of the spectral density and by utilizing Bochner's Theorem [28]:

(16) C(u, v) =σ2(a2v2+1)×  exp(−b2u2a2v2+1),

(17) $$C(u,\;v)=\frac{\sigma^{2}(a|v|+1)}{{\left(a|v|+1\right)}^{2}+b^{2}|u{|}^{2}}$$

(18) C(u, v) = σ2exp(−a|v|−b2|u|2)×exp(−c|v||u|2),

where *a, b *≥ 0 are scaling parameters of time and space, respectively; *c *≥ 0 is an interaction parameter of time and space, and *σ^2 ^*= *C*(0, 0) ≥ 0. Note that when *c *= 0, (18) reduces to a separable model.

Gneiting [27] developed an approach that can produce nonseparable covariance models without relying on Fourier transform pairs. One such model is

(19) C(u, v) =σ2(a|v|2α+1)τ×exp(−b|u|2β(a|v|2α+1)βγ),

with (*u*, *v*) ∈ ℛ × ℛ and where *a, b *> 0 are scaling parameters of space and time, respectively; *α, β *∈ (0, 1] are smoothness parameters of space and time, respectively; *γ *0[1]; *τ *≥ 1/2; and *σ^2 ^*≥ 0. *γ *is a space-time interaction parameter which implies a separable structure when 0 and a nonseparable structure otherwise. Increasing values of *γ *indicates strengthening spatio-temporal interaction.

## Computer Simulation

We investigated the performances of the following nonseparable covariances structures that were presented in the preceding section

(20) C1(u, v) =σ2(a2v2+1)×exp(−b2u2a2v2+1),

(21) $$C_{2}(u,\;v)=\frac{\sigma^{2}(a|v|+1)}{{\left(a|v|+1\right)}^{2}+b^{2}|u{|}^{2}},$$

(22) C3(u, v) =σ2(a|v|+1)×exp(−b|u|(a|v|+1)γ/2),

where a, b ≥ 0; *γ *∈ 0[1] and *σ*^2 ^> 0. *C*_1 _and *C*_2 _correspond to (16) and (17), respectively, and *C*_3 _is a special case of (19) with *α *= 1/2, *β *= 1/2 and *τ *= 1.

We generated photosynthetic rate data using these nonseparable covariances to simulate interaction effects between the two environmental signals in functional mapping of a reaction norm. The generated data was analyzed using the nonparametric estimator Σ*_NP _*proposed by Yap et al. [14] using an *L***_2 _**penalty, and Σ_*AR***(**1**) **_(equation (8)). Note that the underlying covariance structures were very different from the assumed model, Σ_*AR***(**1) _, and we therefore expected to get biased estimates. The issue we wanted to address was the extent to which the bias cannot be ignored and an alternative estimator such as Σ*_NP _*may be more appropriate.

Covariance fit was assessed using entropy (*L_E_*) and quadratic (*L_Q_*) losses:

$$L_{E}(\Sigma ,\hat{\Sigma })=\text{tr}(\Sigma^{-1}\hat{\Sigma })\;-\log{\left|\Sigma^{-1}\hat{\Sigma }\right|}^{\;}-\;m$$

and

$$L_{Q}(\Sigma ,\hat{\Sigma })=\text{tr}{\left(\Sigma^{-1}\hat{\Sigma }\;-I\right)}^{2}$$

where $\hat{\Sigma }$ is the estimate of the true underlying covariance Σ [14,16,29-31]. Each loss function is 0 when $\hat{\Sigma }=\Sigma$ and large values suggest significant bias.

Using a backcross design for the QTL mapping population, we randomly generated 6 markers equally spaced on a chromosome 100 cM long. One QTL was simulated between the fourth and fifth markers, 12 cM from the fourth marker (or 72 cM from the leftmost marker of the chromosome). The QTL had two possible genotypes which determined two distinct mean photosynthetic rate reaction norm surfaces defined by equations (1) and (2) (see also Figure 1). The surface parameters for each genotype were (*α*_1_, *P*_*m*1_(20), *θ*_1_) = (0.02, 2, 0.9) and (*α*_2_, *P*_*m*2_(20), *θ*_2_) = (0.01, 1.5, 0.9). Phenotype observations were obtained by sampling from a multivariate normal distribution with mean surface based on irradiance and temperature levels of {0, 50, 100, 200, 300} and {15, 20, 25, 30}, respectively, and covariance matrix *C***_l_**(*u, v*), l = 1, 2, 3 with *a *= 0.50, *b *= 0.01 for *C*_1_, *a *= 1.00, *b *= 0.01 for *C*_2_, *a *= 1.00, *b *= 0.01, *c *= 0.60 for *C*_3 _and *σ*^2 ^= 1.00 for all three covariances.

**Figure 2:**
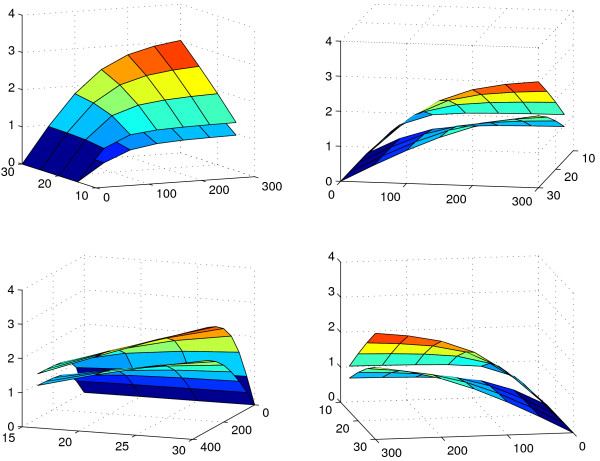
**Reaction norm surfaces of photosynthetic rate as functions of irradiance and temperature**. Models are based on equations (1) and (2) with parameters (*α*_1_, *P*_*m*1_(20), *θ*_1_) = (0.02, 2, 0.9) and (*α*_2_, *P*_*m*2_(20), *θ*_2_) = (0.01, 1.5, 0.9) as used in the simulation.

Figure 2 shows the reaction norm surfaces of photosynthetic rate as functions of irradiance and temperature that were used in the simulation. Within the considered domain of values for irradiance and temperature, one surface lies above the other. These surfaces differ only in terms of the *α*_2 _and *P*_*m*1_(20) parameters.

**Figure 3:**
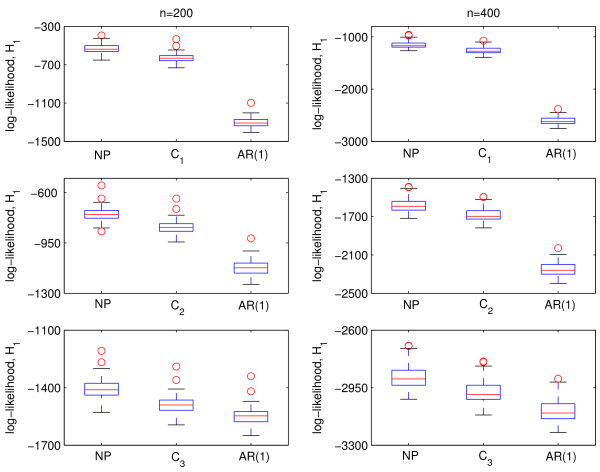
**Boxplots of the values of the log-likelihood under the alternative model, *H*_1_**. Significantly biased estimates by Σ_*AR*(1) _are apparent for *C*_1_.

**Figure 4:**
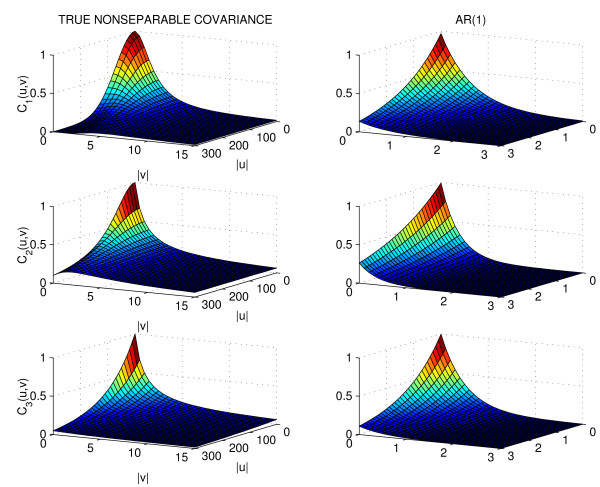
**Covariance plots**. Plots of *C*_l_, *l *= 1, 2, 3 versus irradiance (|*u*|) and temperature (|*v*|) lags are on the left column. On the right column are the estimates of *C*_*l *_by ∑_*AR*(1)_.

**Figure 5:**
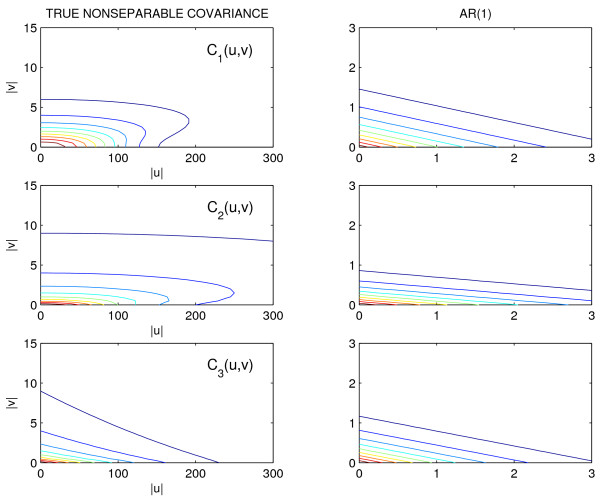
**Contour plots**. Contour plots of *C*_l_, *l *= 1, 2, 3 on the left column. On the right column are the contour plots of the estimates of *C_l _*by Σ_*AR*(1) _.

The functional mapping model was applied to the marker and phenotype data with *n *= 200, 400 samples. The surface defined by equations (1) and (2) was used as mean model with Σ*_NP _*and Σ_*AR*(1**) **_as covariance models to analyze the data generated using *C*_l_(u, v). 100 simulation runs were carried out and the averages on all runs of the estimated QTL location, mean parameter estimates, entropy and quadratic losses, including the respective Monte carlo standard errors (SE), were recorded. Tables 1 and 2 present the results of these simulations. The results show that using Σ*_NP _*yields reasonably accurate and precise parameter estimates. The results for Σ_*AR*(1) _are similar to Σ*_NP _*except that the average losses, given by *L**_E _***and *L**_Q_***, are inflated for *C*_1 _and *C*_2_. Figure 3 shows box plots of the log-likelihood values under the alternative model. These plots reveal biased estimates of *C*_1 _and *C*_2 _by Σ_*AR*(1) _and the degrees of bias are consistent with the average losses. The results for the log-likelihood values under the null model are very similar but are not shown. We also provided the covariance and corresponding contour plots of *C*_l_(*u, v*), *l *= 1, 2, 3 and the Σ_*AR*(1) _estimates of these in Figure 4 and 5. We only provided plots for *C*_l_(*u, v*), *l *= 1, 2, 3 and Σ_*AR*(1) _to illustrate the behavior of these parametric models. We did not include plots for the estimated Σ*_NP _*because there are no parametric estimates for this model and we did not record all elements of the estimated Σ*_NP _*in the simulation runs.

**Table 1:** Averaged QTL position, mean curve parameters, entropy and quadratic losses and their standard errors (given in parentheses) for two QTL genotypes in a backcross population under different sample sizes (*n*) based on 100 simulation replicates (Σ*_NP_*).

		QTL	QTL genotype 1			QTL genotype 2				
						
Covariance	*n*	Location	${\hat{\alpha }}_{1}$	${\hat{P}}_{m1}(20)$	${\hat{\theta }}_{1}$	${\hat{\alpha }}_{2}$	${\hat{P}}_{m2}(20)$	${\hat{\theta }}_{2}$	*L_E_*	*L_Q_*
*C*_1_	200	71.68	0.02	2.02	0.90	0.01	1.52	0.88	1.04	2.03
		(0.28)	(0.00)	(0.01)	(0.00)	(0.00)	(0.02)	(0.01)	(0.01)	(0.02)
	400	72.16	0.02	2.00	0.90	0.01	1.52	0.88	0.53	1.06
		(0.23)	(0.00)	(0.01)	(0.00)	(0.00)	(0.01)	(0.01)	(0.00)	(0.01)
*C*_2_	200	71.88	0.02	2.00	0.90	0.01	1.53	0.88	1.00	1.96
		(0.29)	(0.00)	(0.01)	(0.00)	(0.00)	(0.01)	(0.01)	(0.01)	(0.02)
	400	71.92	0.02	2.00	0.90	0.01	1.52	0.89	0.52	1.02
		(0.17)	(0.00)	(0.01)	(0.00)	(0.00)	(0.01)	(0.01)	(0.00)	(0.01)
*C*_3_	200	72.12	0.02	2.01	0.89	0.01	1.54	0.87	0.88	1.70
		(0.37)	(0.00)	(0.01)	(0.01)	(0.00)	(0.02)	(0.01)	(0.01)	(0.02)
	400	72.08	0.02	2.01	0.90	0.01	1.52	0.89	0.48	0.94
		(0.20)	(0.00)	(0.01)	(0.00)	(0.00)	(0.01)	(0.01)	(0.00)	(0.01)

	**True:**	72.00	0.02	2.00	0.90	0.01	1.50	0.90		

**Table 2:** Averaged QTL position, mean curve parameters, entropy and quadratic losses and their standard errors (given in parentheses) for two QTL genotypes in a backcross population under different sample sizes (*n*) based on 100 simulation replicates (Σ_*AR*(1__)_).

		QTL	QTL genotype 1			QTL genotype 2				
						
Covariance	*n*	Location	${\hat{\alpha }}_{1}$	${\hat{P}}_{m1}(20)$	${\hat{\theta }}_{1}$	${\hat{\alpha }}_{2}$	${\hat{P}}_{m2}(20)$	${\hat{\theta }}_{2}$	*L_E_*	*L_Q_*
*C*_1_	200	72.32	0.02	2.03	0.90	0.01	1.53	0.87	19.43	681.78
		(0.45)	(0.00)	(0.01)	(0.01)	(0.00)	(0.02)	(0.01)	(0.07)	(6.16)
	400	71.72	0.02	2.03	0.90	0.01	1.51	0.89	19.45	684.11
		(0.27)	(0.00)	(0.01)	(0.00)	(0.00)	(0.01)	(0.01)	(0.05)	(4.40)
*C*_2_	200	71.96	0.02	2.01	0.90	0.01	1.55	0.87	4.83	58.60
		(0.34)	(0.00)	(0.01)	(0.00)	(0.00)	(0.02)	(0.01)	(0.02)	(1.01)
	400	71.84	0.02	2.01	0.90	0.01	1.52	0.89	4.83	58.61
		(0.20)	(0.00)	(0.01)	(0.00)	(0.00)	(0.01)	(0.01)	(0.02)	(0.77)
*C*_3_	200	72.00	0.02	2.01	0.89	0.01	1.54	0.87	0.60	1.51
		(0.35)	(0.00)	(0.01)	(0.01)	(0.00)	(0.02)	(0.01)	(0.00)	(0.10)
	400	71.96	0.02	2.01	0.89	0.01	1.52	0.89	0.60	1.43
		(0.22)	(0.00)	(0.01)	(0.00)	(0.00)	(0.01)	(0.01)	(0.00)	(0.08)
										

	**True:**	72.00	0.02	2.00	0.90	0.01	1.50	0.90		

We conducted further simulations using *C*_1 _as the underlying covariance structure of the data with *n *= 400. This was the case where Σ_*AR*(1) _performed the worst. We considered two scenarios: increased variance parameter, *σ*^2^, or increased irradiance and temperature levels (finer grid). That is,

1. *σ*^2 ^= 2, 4 with irradiance and temperature levels of {0, 50, 100, 200, 300} and {15, 20, 25, 30}, respectively.

2. *σ*^2 ^= 1, 2 with irradiance and temperature levels of {0, 50, 100, 150, 200, 250, 300} and {15, 18, 21, 24, 27, 30}, respectively.

We included an analysis of the simulated data using *C*_1 _as the covariance model to ensure the results are not false-positives. The results of the simulation are shown in Tables 3 and 4. The tables include columns for the log-likelihood values under the null (*H*_0_) and alternative (*H*_1_) hypotheses as well as the maximum of the log-likelihood ratio (max*LR*). Max*LR *is used in permutation tests to assess significance of QTL existence (see Section 2.3). Under scenarios (1) or (2), i.e. increased variance parameter *σ*^2 ^or increased irradiance and temperature levels, using Σ*_NP _*yields significantly more accurate and precise estimates of the QTL location compared to Σ_*AR*(1)_: In Table 3, when *σ*^2 ^= 4, the estimates of the true QTL location of 72 were 71.64 and 74.20 for NP and Σ_*AR*(1)_, respectively; In Table 4, when *σ*^2 ^= 2, the estimates were 72.13 and 78.44. Although for Σ_*AR*(1)_, max*LR *appears to be more accurate, the log-likelihood ratios are still significantly different from the estimates given by *C*_1_. Again, this is reflected in the inflated average losses. Note that the max*LR *estimates are larger for Σ_*AR*(1) _when compared to those for Σ*_NP_*. We do not expect this to be always the case. In other instances, the max*LR *estimates for Σ_*AR*(1) _may be smaller than those for Σ*_NP _*. However, in those instances, we expect the max*LR *estimates for Σ*_NP _*to still be more accurate and precise than those for Σ_*AR(*1)_, unless the true underlying covariance structure is Σ_*AR*(1)_, which is not likely.

**Table 3:** Averaged QTL position, mean curve parameters, log-likelihood values, maximum log-likelihood ratios (max*LR*), entropy and quadratic losses and their standard errors (given in parentheses) for two QTL genotypes in a backcross population based on 100 simulation replicates (*C*_1 _with *n *= 400 and *σ*^2 ^= 2, 4).

		QTL	QTL genotype 1			QTL genotype 2			log-likelihood				
								
Covariance	*σ*^2^	Location	${\hat{\alpha }}_{1}$	${\hat{P}}_{m1}(20)$	${\hat{\theta }}_{1}$	${\hat{\alpha }}_{2}$	${\hat{P}}_{m2}(20)$	${\hat{\theta }}_{2}$	*H*_0_	*H*_1_	max*LR*	*L_E_*	*L_Q_*
Σ_*AR*(1)_	2	72.40	0.02	2.05	0.89	0.01	1.52	0.87	-5437	-5373	128.51	19.45	684.37
		(0.44)	(0.00)	(0.01)	(0.01)	(0.00)	(0.02)	(0.01)	(7.36)	(7.31)	(2.45)	(0.05)	(4.44)
	4	74.20	0.02	2.11	0.88	0.01	1.52	0.84	-8175	-8141	65.55	19.44	683.82
		(0.69)	(0.00)	(0.02)	(0.01)	(0.00)	(0.03)	(0.02)	(7.32)	(7.31)	(1.80)	(0.05)	(4.46)
													
*C*_1_	2	71.96	0.02	2.01	0.90	0.01	1.54	0.88	-4088	-4021	133.41	0.01	0.13
		(0.29)	(0.00)	(0.01)	(0.00)	(0.00)	(0.02)	(0.01)	(7.17)	(7.16)	(2.15)	(0.00)	(0.02)
	4	71.96	0.02	2.03	0.89	0.01	1.57	0.86	-6822	-6788	69.07	0.01	0.13
		(0.44)	(0.00)	(0.01)	(0.01)	(0.00)	(0.03)	(0.02)	(7.16)	(7.16)	(1.57)	(0.00)	(0.02)
*N P*	2	72.16	0.02	2.01	0.89	0.01	1.54	0.87	-3967	-3912	109.79	0.53	1.05
		(0.29)	(0.00)	(0.01)	(0.00)	(0.00)	(0.02)	(0.01)	(6.87)	(6.89)	(1.66)	(0.00)	(0.01)
	4	71.64	0.02	2.01	0.89	0.01	1.57	0.84	-6713	-6684	59.92	0.53	1.04
		(0.49)	(0.00)	(0.01)	(0.01)	(0.00)	(0.03)	(0.02)	(6.89)	(6.93)	(1.27)	(0.00)	(0.01)
													

	**True:**	72.00	0.02	2.00	0.90	0.01	1.50	0.90					

**Table 4:** Averaged QTL position, mean curve parameters, log-likelihood values, maximum log-likelihood ratios (max*LR*), entropy and quadratic losses and their standard errors (given in parentheses) for two QTL genotypes in a backcross population based on 100 simulation replicates (*C*_1 _with *n *= 400, increased irradiance and temperature levels, and *σ*^2 ^= 1, 2).

		QTL	QTL genotype 1			QTL genotype 2			log-likelihood				
								
Covariance	*σ*^2^	Location	${\hat{\alpha }}_{1}$	${\hat{P}}_{m1}(20)$	${\hat{\theta }}_{1}$	${\hat{\alpha }}_{2}$	${\hat{P}}_{m2}(20)$	${\hat{\theta }}_{2}$	*H*_0_	*H*_1_	max*LR*	*L_E_*	*L_Q_*
Σ_*AR*(1)_	1	72.16	0.02	2.04	0.90	0.01	1.48	0.88	-1278	-1063	430.01	223	64090
		(0.36)	(0.00)	(0.01)	(0.00)	(0.00)	(0.01)	(0.01)	(14.01)	(14.15)	(4.78)	(0.45)	(261.88)
	2	78.44	0.02	2.15	0.91	0.01	1.48	0.86	-6992	-6876	231.86	222	63923
		(0.84)	(0.00)	(0.02)	(0.00)	(0.00)	(0.02)	(0.01)	(14.08)	(14.16)	(3.62)	(0.44)	(257.89)
													
*C*_1_	1	71.76	0.02	2.01	0.90	0.01	1.51	0.89	4913	5068	309.86	0.01	0.31
		(0.18)	(0.00)	(0.00)	(0.00)	(0.00)	(0.01)	(0.00)	(11.04)	(11.10)	(3.17)	(0.00)	(0.04)
	2	71.76	0.02	2.01	0.90	0.01	1.52	0.88	-821.08	-743.76	154.64	0.01	0.31
		(0.24)	(0.00)	(0.01)	(0.00)	(0.00)	(0.01)	(0.01)	(11.10)	(11.12)	(2.22)	(0.00)	(0.04)
*N P*	1	71.73	0.02	2.01	0.90	0.01	1.51	0.89	5431	5537	212.64	2.34	4.55
		(0.18)	(0.00)	(0.01)	(0.00)	(0.00)	(0.01)	(0.00)	(11.22)	(11.11)	(2.20)	(0.01)	(0.03)
	2	72.13	0.02	2.01	0.90	0.01	1.49	0.89	-336	-273	127.37	2.37	4.53
		(0.34)	(0.00)	(0.01)	(0.00)	(0.00)	(0.01)	(0.01)	(10.44)	(10.42)	(1.72)	(0.01)	(0.03)
													

	**True:**	72.00	0.02	2.00	0.90	0.01	1.50	0.90					

## Discussion

In this paper, we studied the covariance model in functional mapping of photosynthetic rate as a reaction norm to irradiance and temperature as environmental signals. In the presence of interaction between the two signals simulated by nonseparable covariance structures, our analysis showed that Σ*_NP _*is a more reliable estimator than Σ_*AR*(1) _particularly in QTL location estimation. The advantage of Σ*_NP _*over Σ_*AR*(1) _is greater when the variance of the reaction norm process and the number of signal levels increase.

Σ*_NP _*was developed in the context of a one dimensional (longitudinal) vector which has an ordering of variables. The phenotype vector we considered here consists of observations based on two levels of irradiance and temperature measurements, i.e.,

(23) yi=[yi(1,1),...,yi(1,T),︸irradiance 1... ,[yi(S,1),...,yi(S,T)',︸irradiance S

This vector has no natural ordering like in longitudinal data. However, our simulation results still suggest that Σ*_NP _*can be directly applied to observations that have no variable ordering such as (23). The process by which Σ*_NP _*was obtained in Yap et al. [14] was based on non-mixture type of longitudinal covariance estimators. This process is flexible and can potentially accommodate other estimators that can handle unordered data or are invariant to variable permutations. See for example the sparse permutation invariant covariance estimator (SPICE) proposed by Rothman et al. [32].

In the presence of interactions, nonseparable covariances can possibly be used in place of Σ*_NP_*, but they should closely reflect the structure of the data. Unfortunately, as with any parametric model, this is not often the case. In fact, it is not even known whether the data exhibits interactions or not. Before deciding on what model to use, one might utilize tests for separability [33,34]. If separable models are appropriate, then there are many options. Otherwise, it is difficult to choose from a number of complex nonseparable covariances because there are no available general guidelines as yet that can help one decide which model to use. The covariance *C***_3 _**that was used in the simulations had an easy to interpret interaction parameter *γ *∈ 0[1]. However, despite an interaction "strength" of *γ *= 0.6, the separable model, Σ_*AR*(1)_, estimated the data generated by *C*_3 _quite well. Thus, the trade-o between using a nonseparable model instead of a separable one may not be worth it. Another option is to use separable approximations to nonseparable covariances [35]. The nonseparable covariances that we considered were assumed to be stationary and isotropic. These two assumptions may not always hold for real data. Although not specifically addressed here, using Σ*_NP _*may work for data that do not satisfy these assumptions.

Finally, we only considered two environmental signals with interactions: irradiance and temperature. However, the reaction norm of photosynthetic rate is a very complex process because there are really more environmental signals at play other than these two. Theoretically, the spatial domain of spatio-temporal nonseparable covariance models can be extended to more than one dimensions i.e., *d *> 1 in (10). For example, a two dimensional spatial domain models an area on a flat surface while a three dimensional domain models space. There are spatio-temporal models for these. However, this extension cannot be used to increase the number of signals in a reaction norm unless the signals have the same unit of measurement or one assumes separability or no interaction among the signals. For example, carbon dioxide concentration cannot be added as a signal, in addition to irradiance and temperature, when modeling photosynthetic rate as a reaction norm in the functional mapping setting because it does not have the same unit as irradiance or temperature. Thus, it is difficult to simulate data from more than two signals with interactions. However, Σ*_NP _*can theoretically handle covariances associated with more than two signals that may involve interactions. The computer code for the model will be available from http://statgen.psu.edu.

## Authors' contributions

JY participated in the design of the study, performed the statistical analysis, and wrote the manuscript. YL, KD and JL participated in the statistical analysis. RW conceived of the study, participated in its design and coordination, and wrote the manuscript. All authors read and approved the final manuscript.
